# Development and Characterization of Low‐Glycemic Couscous Enriched With β‐Glucans and Proteins

**DOI:** 10.1002/fsn3.72201

**Published:** 2026-08-05

**Authors:** Ece Surek, Kubra Ozkan, Cagla Ozer, Abderrazek Jilal, Ghizlane Salih, Halide Yildirim, Andrea Visioni, Francesco Sestili, Eda Aktas‐Akyildiz, Osman Sagdic, Hamit Koksel

**Affiliations:** ^1^ Institute of Nanotechnology and Biotechnology Istanbul University‐Cerrahpasa Istanbul Türkiye; ^2^ Department of Gastronomy and Culinary Arts, Faculty of Fine Arts, Design and Architecture Istinye University Istanbul Türkiye; ^3^ Department of Food Engineering Yildiz Technical University Istanbul Türkiye; ^4^ National Institute for Agricultural Research Morocco (INRAM) Rabat Morocco; ^5^ Department of Food Technology and Quality National Institute for Agricultural Research of Morocco (INRAM) Rabat Morocco; ^6^ Biodiversity and Crop Improvement Program International Center for Agricultural Research in the Dry Areas Rabat Morocco; ^7^ Department of Agriculture and Forest Sciences (DAFNE) University of Tuscia Viterbo Italy; ^8^ Department of Food Engineering Hitit University Çorum Türkiye

**Keywords:** β‐glucan, antioxidants, couscous, glycemic index, lentils, resistant starch

## Abstract

In this study, a novel functional couscous with reduced glycemic potential was developed by enrichment of traditional couscous with β‐glucan and protein through the addition of hull‐less barley (cv. *Chifaa)* and lentil flours. Couscous samples were produced using 50:50 blends of durum wheat semolina (Svevo) with Chifaa, lentil, or malted lentil flours. The cooking and textural properties, color attributes, and chemical and nutritional composition (protein, β‐glucan, resistant starch, in vitro glycemic index (GI)) were evaluated in both flour blends and couscous products. The incorporation of Chifaa flour significantly improved the technological and nutritional quality of couscous, as well as its phenolic content and antioxidant capacity. Couscous containing Chifaa flour exhibited the highest water absorption, hardness, and chewiness compared to control samples. In particular, the couscous produced from the Chifaa/Lentil blend showed the highest water absorption (279.4%), total phenolic content (448.6 mg GAE/100 g), and total antioxidant capacity (416.6–470.2 mg TE/100 g), as well as the highest β‐glucan and protein contents (3.57% and 22.56%, respectively). Additionally, this sample exhibited the lowest in vitro GI (50.1), meeting the criterion for classification as a low‐GI food (GI < 55) and complying with FDA health claim requirements. These findings demonstrate the potential of barley–legume‐based formulations to develop nutritionally enhanced couscous with improved functional and glycemic properties.

## Graphical abstract

Traditional couscous production with 50/50 blends of different raw materials (wheat, barley and lentil).

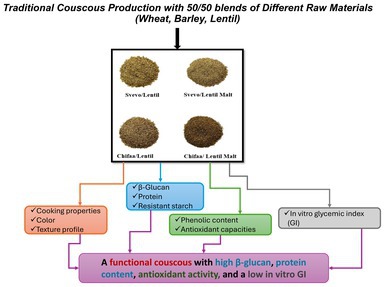

## 1 Introduction

Couscous is an ancient food product with origins dating back to around 2000 years (Abecassis et al. 2012). The term “*couscous*” may have been derived from the Arabic word “*kaskasa*” (to pound small) or from the Berber word “*seksu*” (well‐rolled or well‐formed) (Chemache et al. 2018). Couscous is a steamed and agglomerated product originating from North Africa, typically made by hand using durum wheat semolina. It has gained global recognition due to its ease of production, versatility, and convenience, as well as the increasing trend toward healthier eating and the rising popularity of the Mediterranean diet. In the 17th century, Arabs brought couscous from the Mediterranean basin to Europe, and it eventually made its way to the Americas via Portuguese trade routes from Morocco (Boudouira et al. 2023; Cankurtaran and Bilgiçli 2021; Hammami et al. 2022; Messia et al. 2019). In 2020, UNESCO recognized the knowledge, ancestral expertise, and traditions surrounding the production and consumption of couscous by inscribing them on its list of Intangible Cultural Heritage (UNESCO 2020).

Couscous is traditionally made from durum wheat semolina, but it can also be prepared using semolina blended with various alternative flours as raw materials (Chemache et al. 2021). Several studies have investigated the enrichment of couscous with chickpea flour (Demir et al. 2010; Demir and Demir 2016), fermented acorns, sorghum, and wheat (Belmouloud et al. 2025), bulgur flour (Yuksel et al. 2017), lentil semolina (Benayad et al. 2021; Ibtissame et al. 2022), lentil flour and lentil protein concentrate (Allan et al. 2025), and with other legume‐based flours, including soybean, buckwheat, common bean, and lupine flours (Demir and Demir 2016; Yuksel et al. 2018), to enhance its nutritional composition and functional properties.

Sprouting (or malting) is one of the traditional methods used to improve the nutritional quality in cereals and pulses by reducing antinutritional compounds (Benincasa et al. 2019). Sprouting affects various compositional and nutritional traits of cereals, including the increase in mineral bioavailability, vitamins, and antioxidant compounds (Lemmens et al. 2019). During sprouting, endogenous enzymes such as proteases, amylases, and lipases are activated (Gunathunga et al. 2024). These enzymes hydrolyze complex macromolecules, including proteins, carbohydrates, and lipids, into simpler components, including amino acids, basic sugars, and fatty acids. As a result, nutrient digestibility is enhanced, and the functional properties of cereals and seeds are modified (Benincasa et al. 2019; Marti et al. 2017). Enrichment of couscous with sprouted buckwheat resulted in a product with elevated phenolic compound levels, particularly rutin and quercetin, while also improving textural properties such as cohesiveness, gumminess, and resilience (Giovanelli et al. 2023). Additionally, Moroccan Saharan communities have developed various types of couscous, with one of the most renowned being Couscous El‐Khomassi, which has been prepared for centuries by Saharan housewives from the Laayoun‐Dakhla region. This couscous is highly valued for its rich nutritional profile and exceptional taste, made from a blend of five different flours (bread wheat, barley, roasted barley, roasted corn, and durum wheat) (Zaroual et al. 2019).

The growing demand for functional foods has driven the search for alternative ingredients with superior nutritional properties. Furthermore, food processing has garnered increasing public and scientific interest, largely due to references from international organizations such as the FAO and WHO highlighting the strong association between chronic diseases, lifestyle habits, and unhealthy dietary patterns (Galanakis 2021). Couscous, characterized by a high carbohydrate content and glycemic index, may pose challenges for individuals with diabetes due to its potential impact on glycemic control (Kumar et al. 2022). The glycemic load of couscous can be lowered by reducing portion size, incorporating fiber‐rich ingredients, and selecting alternative garnishes (Kapoor et al. 2024). This study aims to improve an innovative functional couscous made by a women's cooperative from the Souss region in Morocco (Tata province) enriched with different raw materials such as lentil and lentil malt flours as a source of protein and barley flour as a source of β‐glucan, and to evaluate its nutritional, technological, and cooking properties.

## 2 Materials and Methods

### 2.1 Materials

In this research, a durum wheat (cv. Svevo), a hull‐less barley variety with high β‐glucan content (Chifaa), and a high‐protein lentil genotype were used as raw materials for the production of couscous. The lentils were used in raw and malted forms.

### 2.2 Preparation of Flour and Couscous Samples

Lentil malt was prepared through four steps. Firstly, lentils were soaked in water at ambient temperature for 8 h. After draining the water, the lentils were left to sprout for 2 days. The sprouted lentils were then dried at 35°C for 48 h to preserve their nutritional properties. Once they dried, the lentil malt was mixed with other grains (Svevo or Chifaa) before grinding to provide a uniform flour for couscous production. Raw (unmalted) lentils were also mixed with other grains before grinding, without any prior soaking or drying.

The limitation of this study is that each formulation was produced in a single batch, and three subsamples were randomly taken from each couscous sample for laboratory analyses. Therefore, inter‐batch variability among couscous samples was not assessed. Analytical determinations were performed on triplicate subsamples to ensure measurement precision. Accordingly, the statistical analysis reflects analytical variability rather than inter‐batch production variability.

**FIGURE 1:**
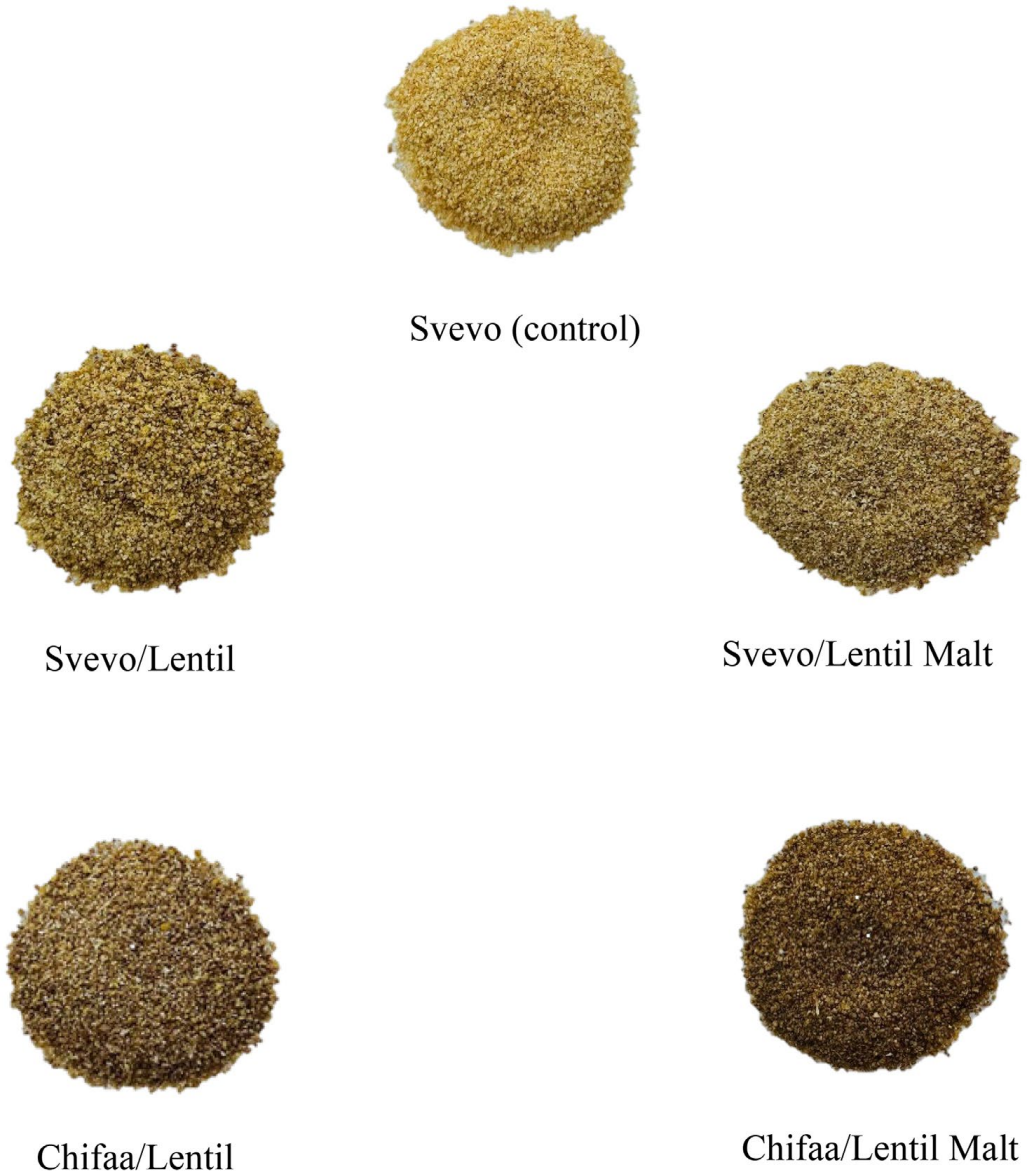
Couscous samples produced with 100% Svevo (control), Svevo/Lentil, Svevo/Lentil Malt, Chifaa/Lentil, and Chifaa/Lentil Malt flour blends.

All wheat and barley grains were carefully cleaned. The following innovative formulations were developed to diversify the range and enhance the nutritional quality of samples: couscous with 50% lentil‐50% Chifaa mixture flour, couscous with 50% lentil‐50% Svevo mixture flour, couscous with 50% lentil malt‐50% Chifaa mixture flour, and couscous with 50% lentil malt‐50% Svevo mixture flour (Figure 1). Additionally, couscous produced from 100% Svevo durum wheat semolina was used as the control sample. The 50/50 blends were prepared and ground in a Brabender (Quadrumat Junior, Duisburg, Germany) laboratory mill. It was possible to develop samples enriched in both β‐glucan and protein by using the 50:50 substitution as reported by Arer et al. (2025). The couscous was traditionally handmade by experienced women from a women's cooperative located in the southwest of Morocco. The production of couscous followed the traditional manufacturing process, which was a meticulous artisanal method: Firstly, the flour was moistened with water and manually rolled to form couscous granules. The freshly prepared couscous was then steamed for 2 h to ensure good preservation and reduce preparation time for households. After steaming, the couscous was spread out and air‐dried for 3 days. Then the dry couscous was packaged to ensure the conservation and distribution of the final product (Abecassis et al. 2012; Benayad et al. 2021).

### 2.3 Analysis of Cooking Properties of Couscous Samples

The optimum cooking time for couscous samples was determined by the method explained by Tekin‐Cakmak et al. (2024). Cooking properties such as water absorption (%), volume increase (%), cooking loss (%), and total organic matter (g/100 g) of couscous samples, which are presented in Table 1, were determined as described by Arer et al. (2025). Briefly, 10 g of the couscous sample was weighed (W1) and placed into a measuring cylinder containing 100 mL of distilled water. After the increase in water volume was noted (V1), the samples were cooked with 100 mL of boiling water. The cooked couscous samples were drained using wire mesh strainer, and the cooking water was placed into a beaker, which was tared before, and dried in an oven at 100°C overnight (Khouryieh et al. 2006). The residue after drying was weighed, and the cooking loss was calculated as the percentage of the weight of the starting sample. After the cooked couscous samples were weighed (W2) and placed into 100 mL of distilled water to measure the increase in water level (V2), the cooked samples were used for analysis of total organic matter (TOM), which is the surface material released from cooked product during rinsing (Basman and Yalcin 2011), as described by Tekin‐Cakmak et al. (2024) and D'Egidio et al. (1982). The results were expressed as the gram amount of starch transferred from the 100 g sample to the washing water of the cooked couscous sample (Tekin‐Cakmak et al. 2024). The water absorption (%) and volume increase (%) values of couscous samples were determined by calculating the differences between W2 and W1; V2 and V1 as the percentages of W1 and V1, respectively.

**TABLE 1:** Cooking properties of couscous samples.

	Water absorption (%)	Volume increase (%)	Cooking loss (%)	Total organic matter (g/100 g)
Couscous*				
Svevo/Lentil	252.0 ± 9.90^ab^	426.9 ± 16.32^a^	9.61 ± 0.49^b^	2.33 ± 0.05^b^
Svevo/Lentil Malt	216.0 ± 8.49^b^	364.0 ± 33.94^ab^	9.30 ± 0.91^b^	2.79 ± 0.24^ab^
Chifaa/Lentil	279.4 ± 6.74^a^	323.3 ± 23.57^b^	9.70 ± 0.97^b^	2.62 ± 0.14^ab^
Chifaa/Lentil Malt	220.5 ± 2.64^b^	385.0 ± 21.21^ab^	15.00 ± 0.74^a^	3.73 ± 0.29^a^

*Note:* Data are expressed as mean ± standard deviation; mean values in each column with different letters (a–d) are significantly different (*p <*  0.05).

* The cooking properties of the 100% Svevo control couscous were not determined. Svevo or Chifaa was mixed with Lentil or Lentil Malt in equal proportions before grinding.

### 2.4 Characterization of Flour and Couscous Samples

The moisture contents of flour and couscous samples were analyzed according to AACC Method No 44‐15A, and total nitrogen contents of samples were determined according to AACC Methods No 46‐10.0 (protein = 6.25 × *N*) (AACC 2010).

The β‐glucan contents of flour and couscous samples were determined by an enzymatic method using the β‐glucan Assay Kit, and the amounts of enzyme‐resistant starch in samples were measured using the Resistant Starch Assay Kit (Megazyme International Ireland Ltd., Ireland).

Color measurements of flour and couscous samples were carried out using a colorimeter (CR‐400, Konica Minolta, Tokyo, Japan). The colorimeter was calibrated to a white calibration plate before use. The color values were determined according to the CIELAB color space system. Color was expressed as L* (lightness/darkness; 100, white; 0, black), a* (redness/greenness; +, red; −, green), and b* (yellowness/blueness; +, yellow; −, blue).

### 2.5 Determination of Total, Insoluble, and Soluble Dietary Fiber Contents

The total dietary fiber (TDF), insoluble dietary fiber (IDF), and soluble dietary fiber (SDF) contents of the couscous samples were determined by the enzymatic‐gravimetric method using a Megazyme assay kit (AACC Method No. 32–07.01, AACC 2010).

### 2.6 Determination of in Vitro Glycemic Index Value

In vitro glycemic index (GI) values of cooked couscous samples were determined using the D‐Glucose Assay Kit (GOPOD Format) (Megazyme International Ireland Ltd., Ireland) following the protocols described by Goni and Saura‐Calixto (1997) and Tekin‐Cakmak et al. (2024).

### 2.7 Texture Profile Analysis of Couscous Samples

Texture properties of cooked couscous samples were measured using a Texture Analyzer (TA.XT2 Plus, Stable Micro System Ltd., Surrey, UK) according to the method by Tekin‐Cakmak et al. (2024). Fifteen grams of cooked couscous samples were placed in a metal ring (60 mm in diameter and 30 mm in height) positioned at the center of the testing platform to ensure uniformity in shape across all samples. The texture profile attributes assessed included hardness, adhesiveness, springiness, cohesiveness, chewiness, and resilience.

### 2.8 Determination of Phenolic Contents and Antioxidant Capacities of Flour and Couscous Samples

The free and bound phenolic compounds of the flour and couscous samples were extracted according to Shamanin et al. (2022). The extracts were stored at −18°C until analysis. The concentrations of free and bound phenolics were measured using the Folin–Ciocalteu method (Singleton and Rossi 1965), and the results were given as mg of gallic acid equivalent (GAE) per 100 g of dry weight (dw). The total phenolic content concentration (TPC) was calculated as the sum of the concentrations of free and bound phenolics. ABTS, DPPH, and FRAP antioxidant capacities of samples were determined by a spectrophotometer (Shimadzu 150 UV‐1800, Kyoto, Japan) according to the methods described in Re et al. (1999), Singh et al. (2002), and Benzie and Strain (1996), respectively. The results were reported as mg Trolox equivalent (TE) per 100 g dw.

### 2.9 Statistical Analysis

All experiments were implemented in triplicate, and the results were presented as the mean ± standard deviation. Statistical analysis was conducted using SPSS Statistics Software (IBM version 21, USA). One‐way ANOVA followed by Tukey's post hoc test was applied to evaluate the statistical differences among the couscous samples (α = 0.05).

## 3 Results and Discussion

### 3.1 Cooking Properties of Couscous Samples

Cooking properties are among the most important couscous quality attributes affecting consumer acceptance (Demir and Demir 2016). Water absorption (WA), volume increase (VI), cooking loss (CL), and total organic matter (TOM) values of the samples are presented in Table 1. Chifaa/Lentil and Svevo/Lentil couscous samples exhibited the highest WA values (279.4% and 252.0%, respectively), compared with the couscous samples containing lentil malt (216.0% and 220.5%). Notably, replacing non‐malted lentil with malted lentil in the Chifaa‐based formulation caused a significant decrease in WA, from 279.4% to 220.5%.

In contrast to WA, VI followed a different trend. The highest VI value was observed in the Svevo/Lentil sample (427%), which was significantly higher than that of the Chifaa/Lentil sample (323%). In addition, incorporation of lentil or lentil malt into Svevo‐ or Chifaa‐based blends did not result in significant differences in VI values. These findings are consistent with the fact that swelling behavior in couscous is strongly influenced by the raw materials used during production (Messia et al. 2019), and higher swelling values are generally associated with superior product quality (Boudouira et al. 2023). From a practical perspective, good‐quality couscous should rapidly absorb sauce while maintaining structural firmness (Boudouira et al. 2023).

Unlike other pasta products, couscous does not undergo kneading or extrusion; instead, hydration followed by steaming is applied prior to drying. This processing sequence promotes extensive starch gelatinization, which contributes to higher WA upon rehydration during cooking (Messia et al. 2019). Therefore, WA is considered a critical quality parameter for couscous (Allan et al. 2025) and plays a major role in determining the texture of cooked pasta products (Demir and Demir 2016).

Several factors, including starch and fiber contents, the strength of the protein network, and optimum cooking time, can influence water uptake in pasta (Boudouira et al. 2023). Benayad et al. (2021) reported that wheat couscous enriched with lentil semolina exhibited higher WA than wheat semolina couscous, which was attributed to the higher fiber and protein contents of lentils. Similarly, Demir and Demir (2016) produced couscous using different ratios of legume flours and observed that WA significantly increased from 149% to 218% (*p* < 0.05), while VI remained unchanged (144–145%). However, Allan et al. (2025) reported a negative correlation between protein concentration and WA, suggesting that increasing lentil protein concentration reduced starch content and thus decreased the number of available hydroxyl groups for moisture absorption. In line with this explanation, Demir et al. (2010) showed that substituting wheat flour with chickpea flour significantly decreased WA and VI; compared with the control, WA and VI decreased by 13% and 23%, respectively, in couscous containing 50–100% chickpea flour. Moreover, as the level of chickpea increased, VI decreased significantly (from 160 to 106), likely due to reduced starch content and consequently lower swelling capacity.

Overall, WA and VI values of couscous samples containing lentil or lentil malt in this study were higher than those reported for traditional couscous (WA: 140–194%; VI: 158–175%) in previous studies (Cankurtaran Kömürcü 2025; Cankurtaran and Bilgiçli 2021). Likewise, Cankurtaran Kömürcü (2025) reported WA values of 151–188% and VI values of 227–241% in couscous enriched with 20% pea or mung bean flour (protein contents 16% and 15%, respectively).

During cooking, soluble components such as proteins, amylose, and non‐starch polysaccharides may leach into the cooking water (Moayedi et al. 2021). Cooking loss (CL) is therefore regarded as an important quality parameter for pasta and related products and a reliable indicator of cooking performance (Demir and Demir 2016). Higher CL is generally associated with lower product quality and greater stickiness (Majzoobi et al. 2011). In the present study, CL values ranged from 9.30% to 9.70% for most samples; however, the Chifaa/Lentil Malt blend showed a markedly higher CL value (15%). These CL values were higher than those reported for traditional couscous (3.5%–6.0%) (Cankurtaran Kömürcü 2025; Cankurtaran and Bilgiçli 2021). Consistent with this, previous studies have shown that the incorporation of legumes (pea, lentil, chickpea) increases CL as legume addition disrupts the structure of the matrix and facilitates solute leaching (Krishnan and Prabhasankar 2012). For example, Cankurtaran Kömürcü (2025) reported CL values of 5.7% and 6.3% in couscous, including 20% pea and mung bean flour, respectively, while Demir and Demir (2016) reported CL of 6.6% for lentil couscous compared with 4.2% for the control used in their study (100% wheat flour). Demir et al. (2010) also observed a significant increase in CL (5.90–8.15) in chickpea‐enriched couscous compared with the control (5.50), which they attributed to reduced gelatinizable starch. Similarly, Messia et al. (2019) reported higher CL (17%) in barley‐enriched couscous (mixtures of 70% semolina and 30% barley coarse fraction) compared with control semolina couscous (11.5%). Overall, addition of legumes might have a weakening effect on the cohesion of particles forming couscous agglomerates, increase disintegration and consequently intensify CL (Boudouira et al. 2023).

Similar to CL, TOM theoretically reflects the amount of starch and other biochemical components released from the pasta–protein matrix and lost to the cooking medium (Krishnan and Prabhasankar 2012). In this study, the highest TOM value was observed in Chifaa/Lentil Malt (3.73%), consistent with its high CL. However, this value differed significantly only from that of the Svevo/Lentil sample (2.33%). Malting did not significantly affect TOM values. D'Egidio et al. (1982) proposed TOM thresholds to classify spaghetti quality (very high quality: < 1.4 g/100 g; good quality: 1.4–2.1 g/100 g; poor quality: > 2.1 g/100 g). Although all TOM values in the present study exceeded 2.1 g/100 g and would therefore fall within the “poor quality” category under these criteria, it should be emphasized that these thresholds were developed for spaghetti‐type pasta. Because couscous processing includes a steaming step that promotes extensive starch gelatinization, higher TOM values are expected; therefore, spaghetti‐based TOM thresholds should not be directly applied to couscous. Nevertheless, TOM remains a useful comparative indicator for evaluating differences among couscous formulations.

In line with this interpretation, Belleggia et al. (2016) reported the highest TOM value (2.85%) in pasta enriched with 10% debranning fractions of common wheat, likely due to disruption of the gluten network by bran inclusion. Nocente et al. (2019) similarly reported that enriching pasta with barley spent grain (10–20 g/100 g) increased TOM, which was associated with reduced pasta quality. They attributed this effect to the high fiber content of barley spent grain, which can disrupt the starch–gluten network, promote swelling of starch granules, and increase starch release during cooking.

### 3.2 Color Properties of Couscous Samples

The color profile of couscous is a key quality attribute influencing consumer acceptability and preferences (Belmouloud et al. 2025). In particular, consumers generally prefer couscous with a bright yellow appearance (Demir et al. 2010). Yellow coloration is mainly associated with pigment content and enzymatic activity (lipoxygenase), whereas redness is typically related to the formation of Maillard reaction products during thermal processing (Oliver et al. 1993). Because the couscous preparation process used in this study did not involve high‐temperature treatments, the samples were expected to exhibit relatively lower a* values and higher L* values. Moreover, L* values above 60 have been reported as desirable for couscous (Charles et al. 2007). Overall, the color results obtained in this study were consistent with these expectations. Among the flour samples (Table 2), Chifaa flour showed the highest lightness (L* = 88), whereas Lentil Malt flour exhibited the lowest (L* = 76). The L* values of Svevo and Lentil flours were not significantly different from each other; however, Lentil Malt flour was clearly darker than the raw Lentil flour. In contrast to the trends observed for L* and b*, malting increased the a* value of lentil flour, indicating increased redness, while significantly decreasing brightness and yellowness. In addition, a* values differed significantly among flours (*p* < 0.05): the highest a* value was found in Svevo flour (1.87), whereas the lowest was observed in Lentil flour (−1.37). The highest b* value was recorded for Lentil flour (30.10), while Chifaa flour showed the lowest b* value (13.27), which was substantially lower than those of the other flours. The b* value of Lentil Malt flour was not significantly different from that of Svevo (Table 2). Compared with the Chifaa flour previously used for bulgur production, Chifaa flour used in this study presented slightly lower L* and higher a* and b* values (Tekin‐Cakmak et al. 2024).

**TABLE 2:** Color values of couscous samples.

	L*	a*	b*
Flour			
Svevo	79.74 ± 0.60^b^	1.87 ± 0.24^a^	25.30 ± 0.52^b^
Chifaa	88.28 ± 0.28^a^	0.97 ± 0.08^b^	13.27 ± 0.71^c^
Lentil	80.33 ± 0.49^b^	−1.37 ± 0.03^d^	30.10 ± 0.69^a^
Lentil Malt	76.53 ± 0.34^c^	0.49 ± 0.06^c^	26.89 ± 0.76^b^
Couscous*			
Svevo (control)	75.60 ± 0.01^A^	2.60 ± 0.02^C^	24.04 ± 0.13^A^
Svevo/Lentil	74.00 ± 0.30^B^	1.36 ± 0.03^D^	21.16 ± 0.16^C^
Svevo/Lentil Malt	70.58 ± 0.45^D^	2.70 ± 0.10^B^	23.32 ± 0.33^B^
Chifaa/Lentil	72.19 ± 0.05^C^	2.83 ± 0.03^B^	18.65 ± 0.32^E^
Chifaa/Lentil Malt	68.25 ± 0.38^E^	3.38 ± 0.04^A^	19.42 ± 0.08^D^

*Note:* Data are expressed as mean ± standard deviation; mean values in each column with different letters (a–d, A–D) are significantly different (*p <*  0.05). Grain samples (wheat, lentil, and barley, a–d) and couscous samples (A–D) were evaluated separately.

* Svevo or Chifaa was mixed with Lentil or Lentil Malt in equal proportions before grinding.

For couscous samples, substituting lentil malt for lentil in the blends caused significant increases in a* and b* values and a significant decrease in L* values for both the Svevo‐ and Chifaa‐based formulations (*p* < 0.05). Accordingly, the relationship observed between Lentil and Lentil Malt flours for L* and a* was also reflected in the couscous samples produced from these blends. Interestingly, although Lentil Malt flour had a lower b* value than Lentil flour, couscous prepared from Svevo/Lentil Malt and Chifaa/Lentil Malt exhibited higher b* values than the corresponding Svevo/Lentil and Chifaa/Lentil samples, respectively. Overall, L* and b* values differed among all couscous samples. The highest L* (75.60) and b* (24.04) values were observed in 100% Svevo (control) couscous. Regarding a*, Chifaa/Lentil Malt couscous exhibited the highest value (3.4), whereas Svevo/Lentil had the lowest (1.4). The a* values of Svevo/Lentil Malt and Chifaa/Lentil couscous samples were not significantly different (*p* > 0.05).

Messia et al. (2019) reported that incorporation of barley flour into couscous formulation resulted in lower L* and b* values but higher a* values compared with control couscous produced from 100% semolina. Consistent with this trend, couscous samples containing Chifaa (as a β‐glucan source) exhibited lower L* and b* values and higher a* values than Svevo‐based couscous samples. Furthermore, gluten‐free couscous produced from lentil–rice flour mixtures by Boudouira et al. (2023) showed lower L* (59.93–66.61) and higher a* (4.57–4.70) and b* (26.17–27.88) values compared with those observed in the present study. The L* and b* values of all couscous samples in this study were also consistent with those reported for semolina‐based couscous by Aboubacar and Hamaker (1999). Conversely, other studies have reported higher L* (Çelik et al. 2004; Demir et al. 2010) and a* (Belmouloud et al. 2025; Çelik et al. 2004), or lower L* (Belmouloud et al. 2025; Cankurtaran Kömürcü 2025), a* (Cankurtaran Kömürcü 2025; Demir et al. 2010), and b* values (Belmouloud et al. 2025) in traditional couscous or couscous produced from alternative raw materials such as chickpea or fermented cereals compared with the values obtained in the present study. These variations may arise from differences in flour type and proportion, the phenolic profile of the raw materials, and processing conditions, particularly drying, where lower drying temperatures can reduce carotenoid degradation and consequently increase L* values (Abecassis et al. 2012; Aboubacar and Hamaker 1999; Demir et al. 2010).

### 3.3 Protein, β‐Glucan, Resistant Starch, Dietary Fiber Contents, and in Vitro GI of Flour and Couscous Samples

The chemical composition (β‐glucan, resistant starch, dietary fiber, and protein contents) and in vitro glycemic index (GI) values of flour and couscous samples are presented in Table 3. In the present study, the β‐glucan content, resistant starch, and protein contents of the 100% Svevo (control) sample were found to be 0.80%, 1.05%, and 12.71%, respectively. The results clearly demonstrate that the type of raw material has a major impact on the β‐glucan, protein, and resistant starch contents of both the flours and the couscous samples produced from them. Similarly, Messia et al. (2019), who characterized commercial couscous samples obtained from different raw materials such as durum wheat, rice, and barley, reported protein contents ranging from 6.2% to 16.1% and β‐glucan contents between 2.79% and 4.09%, concluding that raw material selection plays a decisive role in determining couscous composition.

**TABLE 3:** The chemical analysis of the flour and couscous samples.

	Samples	β‐Glucan (g/100 g dw)	Protein content	Resistant starch (%)	TDF (%)	IDF (%)	SDF (%)	Glycemic index (GI)
Flour	Svevo	0.58 ± 0.01^b^	15.19 ± 0.28^c^	0.36 ± 0.02^d^				
	Chifaa	7.26 ± 0.03^a^	19.03 ± 0.42^b^	0.45 ± 0.02^c^				
	Lentil	0.06 ± 0.00^c^	26.41 ± 0.40^a^	2.19 ± 0.04^b^				
	Lentil Malt	0.03 ± 0.00^c^	26.75 ± 0.20^a^	2.30 ± 0.02^a^				
Couscous *	Svevo (control)	0.80 ± 0.01^C^	12.71 ± 0.24^C^	1.05 ± 0.06^B^	19.46 ± 0.17^D^	16.06 ± 0.09^C^	3.40 ± 0.08^C^	67.47 ± 0.15^A^
	Svevo/Lentil	0.37 ± 0.04^D^	20.60 ± 0.12^B^	1.78 ± 0.02^A^	25.29 ± 0.18^A^	20.21 ± 0.47^A^	5.09 ± 0.30^A^	60.14 ± 0.50^C^
	Svevo/Lentil Malt	0.36 ± 0.01^D^	20.93 ± 0.14^B^	1.82 ± 0.03^A^	24.88 ± 0.34^AB^	20.77 ± 0.43^A^	4.11 ± 0.09^bc^	66.27 ± 0.36^B^
	Chifaa/Lentil	3.57 ± 0.10^A^	22.50 ± 0.28^A^	1.85 ± 0.03^A^	23.02 ± 0.00^C^	18.30 ± 0.15^B^	4.74 ± 0.15^AB^	50.12 ± 0.09^E^
	Chifaa/Lentil Malt	3.11 ± 0.11^B^	22.56 ± 0.40^A^	1.88 ± 0.02^A^	24.29 ± 0.23^AB^	19.66 ± 0.54^AB^	4.63 ± 0.31^AB^	53.77 ± 0.09^D^

*Note:* Data are expressed as mean ± standard deviation, mean values in each column with different letters (a–d, A–D) are significantly different (*p* < 0.05). Grain samples (wheat, lentil, and barley, a–d) and couscous samples (A–D) were evaluated separately.

* Svevo or Chifaa was mixed with Lentil or Lentil Malt in equal proportions before grinding.

Among the flours analyzed, Chifaa flour exhibited a markedly higher β‐glucan content (7.26%) than Svevo, Lentil, and Lentil Malt flours, whose β‐glucan levels ranged from 0.03% to 0.58%. The β‐glucan content of Chifaa flour was consistent with previously reported values (6.95%–7.05%) (Romano et al. 2022; Tekin‐Cakmak et al. 2024) and with that of micronized barley flour (7.9%) reported by Messia et al. (2019). Romano et al. (2022) reported a slightly lower β‐glucan content for Svevo flour (0.49%) compared to the value obtained in the present study (0.58%). Moreover, malting did not result in significant differences in β‐glucan or protein contents of lentil flour (*p* > 0.05). A similar trend was observed in couscous samples, where significantly higher β‐glucan contents were found in formulations prepared with hull‐less barley Chifaa blends (3.11% and 3.57%) compared with those prepared using Svevo blends (0.36% and 0.37%) (*p* < 0.05).

The Food and Drug Administration (FDA) has authorized a health claim for whole‐grain barley and certain dry‐milled barley products as sources of β‐glucan soluble fiber (FDA 2005). According to FDA guidelines, foods should provide at least 0.75 g of β‐glucan per serving, and a daily intake of at least 3 g of β‐glucan (assuming four eating occasions per day) is recommended to effectively lower serum cholesterol and reduce the risk of coronary heart disease (FDA 2005). Based on USDA FoodData Central USDA (2019), one cup of cooked couscous corresponds to 157 g. Accordingly, couscous samples supplemented with 50% Chifaa barley flour in the present study could provide approximately 3.1–3.6 g of β‐glucan with a consumption of 200 g of cooked couscous (assuming a moisture content of ~50%), thereby meeting the daily β‐glucan intake required for the authorized health claim. Thus, incorporating barley into the diet through couscous consumption may represent a practical strategy to achieve recommended β‐glucan intake and associated health benefits.

Lentil and Lentil Malt flours exhibited significantly higher protein contents (> 26%) than Svevo and Chifaa flours (approximately 15% and 19%, respectively) (*p* < 0.05). The Chifaa/Lentil and Chifaa/Lentil Malt formulations had a higher protein content than the corresponding Svevo/Lentil and Svevo/Lentil Malt samples. The higher protein content observed in lentil‐enriched couscous is consistent with the high protein concentration of lentil flour. In addition, lentil proteins are known to be rich in lysine, which may complement the amino acid profile of durum wheat‐based products (Shevkani et al. 2024). Notably, the Chifaa/Lentil couscous sample, characterized by higher β‐glucan and protein contents, also exhibited the highest water absorption value, as discussed in section 3.1. Consistent with this observation, Messia et al. (2019) reported that functional couscous enriched with β‐glucan‐rich barley flour showed increased water absorption and swelling due to the high water‐holding capacity of β‐glucans.

Overall, the protein contents of couscous samples in the present study (20.60%–22.56%) were higher than those reported for traditional or commercial couscous (9.5%–13.95%) (Burbano‐Agreda et al. 2023; Cankurtaran Kömürcü 2025; Carcea et al. 2017), as well as for gluten‐free couscous containing field bean, chickpea, or lentil flours (10.39%–15.93%). Comparable protein values were reported by Cankurtaran Kömürcü (2025) for couscous enriched with 20% pea or mung bean flour (16.12% and 14.99%, respectively). In contrast, even higher protein contents have been reported when larger proportions of legume flours were used. For example, Benayad et al. (2021) reported protein contents ranging from 19.97% to 23.67% in lentil‐enriched couscous, while Demir et al. (2010) achieved a protein content of 18.4% using 100% chickpea flour.

The resistant starch contents of the flours differed significantly from each other (*p* < 0.05), whereas no significant differences were detected among the couscous samples prepared from their blends (Table 3). Lentil and Lentil Malt flours exhibited higher resistant starch contents (2.19% and 2.30%, respectively) than Svevo and Chifaa flours (0.36% and 0.45%). These findings are consistent with the resistant starch content reported for lentil flour (2.12%) by Romano et al. (2022). Differences in resistant starch levels may be attributed to the presence of protein–starch complexes and polyphenols, which can inhibit enzymatic starch digestion (Khemiri et al. 2022). Dodi et al. (2023) reported lower resistant starch contents in couscous (0.40%) and slightly higher values (0.50%–0.68%) in other pasta products such as spaghetti. Conversely, Khemiri et al. (2022) reported higher resistant starch contents (2.99%–3.49%) in couscous enriched with algae, likely reflecting differences in biochemical composition and formulation.

Total dietary fiber (TDF), insoluble dietary fiber (IDF), and soluble dietary fiber (SDF) contents of control sample were 19.46%, 16.06%, and 3.40%, respectively. Incorporation of lentil flour and lentil malt flour significantly (*p* < 0.05) increased dietary fiber contents of couscous samples compared with those of the control. The highest TDF (25.29%) and SDF (5.09%) contents were observed in Svevo/Lentil sample, whereas the highest IDF content (20.77%) was observed in the Svevo/Lentil Malt sample. The Chifaa/Lentil and Chifaa/Lentil Malt samples also exhibited significantly higher dietary fiber contents than the control; however, their TDF and IDF values were slightly lower than those of corresponding Svevo blends.

The optimum cooking time was determined as 3 min for Chifaa/Lentil, Chifaa/Lentil Malt, and Svevo/Lentil couscous samples, and 2 min for the Svevo/Lentil Malt sample. In vitro GI values ranged from 50.12 to 66.27 (Table 3), with significant differences among samples (*p* < 0.05). According to ISO guidelines, foods are classified as low GI (≤ 55), medium GI (56–69), or high GI (≥ 70) (ISO 2010). The incorporation of Chifaa flour into Lentil and Lentil Malt couscous resulted in GI values of 50.12 and 53.77, respectively, which meet the ISO criterion for low‐GI foods, suggesting the potential for the development of functional couscous with a lower GI; however, GI should also be validated in human studies because the methodology applied in vitro studies has limitations. In contrast, couscous samples prepared with 100% Svevo (control), Svevo and Lentil or Lentil Malt blend exhibited higher GI values (67.47, 60.14, and 66.27), corresponding to the medium‐GI category.

Notably, couscous samples containing Chifaa flour combined lower GI values with higher β‐glucan contents (3.1%–3.6%), suggesting that β‐glucan enrichment played a key role in reducing glycemic response. β‐Glucans derived from oats and barley have been widely reported to attenuate postprandial glycemic responses when incorporated into foods (Aldughpassi et al. 2012). Similarly, Chillo et al. (2011) demonstrated that the addition of 10% barley concentrate to fresh pasta significantly increased fiber content and reduced postprandial GI from 64 to 29. Turco et al. (2019) reported very low GI values (20.07–23.39) for pasta produced entirely from legume flours. Di Pede et al. (2021) further confirmed that pasta is generally classified as a medium‐ to low‐GI food, although substantial variability exists depending on formulation. In comparison, couscous has been reported to exhibit slightly higher GI values (60–65) than pasta (50–55) (Hammami et al. 2022).

Studies focusing specifically on the GI of couscous remain limited. The addition of algae was shown to significantly reduce the estimated GI value of couscous from 61.50 to 54.75; this is consistent with the GI values obtained for Chifaa‐containing couscous samples in the present study. These variations were attributed to differences in biochemical composition. Given that high‐GI foods are associated with increased risk of chronic diseases such as type 2 diabetes, cancer, and cardiovascular diseases, the development of nutritionally enhanced, low‐GI couscous products represents an important target for the food industry, particularly for specific consumer groups such as individuals with type 2 diabetes (Di Pede et al. 2021).

### 3.4 Textural Properties of Cooked Couscous Samples

Textural properties are among the most important factors influencing the overall quality and consumer acceptance of pasta and related products (Cankurtaran Kömürcü 2025). High‐quality couscous is generally expected to exhibit a soft appearance and be easy to chew after rehydration (Hammami et al. 2022). Accordingly, the textural properties of couscous samples, including hardness, adhesiveness, springiness, cohesiveness, chewiness, and resilience, were evaluated in this study (Table 4).

**TABLE 4:** Textural properties of the cooked couscous samples.

	Sample	Hardness (N)	Adhesiveness	Springiness	Cohesiveness	Chewiness	Resilience
Couscous*	Svevo (control)	11.72 ± 1.15^c^	−1.70 ± 0.05^b^	0.85 ± 0.01^b^	0.55 ± 0.01^a^	6.66 ± 0.13^c^	0.43 ± 0.01^a^
	Svevo/Lentil	15.53 ± 1.43^b^	−2.54 ± 0.42^c^	0.92 ± 0.01^a^	0.53 ± 0.03^a^	6.81 ± 0.54^b^	0.36 ± 0.03^b^
	Svevo/Lentil Malt	16.39 ± 1.28^b^	−2.15 ± 0.09^c^	0.93 ± 0.03^a^	0.52 ± 0.02^a^	6.75 ± 0.04^b^	0.37 ± 0.04^b^
	Chifaa/Lentil	19.58 ± 1.01^a^	−0.73 ± 0.12^a^	0.92 ± 0.02^a^	0.58 ± 0.01^a^	8.78 ± 0.18^a^	0.35 ± 0.02^b^
	Chifaa/Lentil Malt	16.74 ± 0.93^ab^	−0.79 ± 0.18^a^	0.83 ± 0.03^b^	0.53 ± 0.02^a^	7.04 ± 0.43^b^	0.27 ± 0.02^c^

*Note:* Data are expressed as mean ± standard deviation; mean values in each column with different letters (a–d) are significantly different (*p* < 0.05).

* Svevo or Chifaa was mixed with Lentil or Lentil Malt in equal proportions before grinding.

Among the samples, the couscous produced from the Chifaa/Lentil blend exhibited the highest hardness value (19.58 N); however, this value was not significantly different (*p* > 0.05) from that of the Chifaa/Lentil Malt sample (16.74 N). Incorporation of Lentil Malt into either Chifaa or Svevo formulations did not result in significant differences in hardness values (16.74 and 16.39 N, respectively). In contrast, when Lentil flour was used, blending with Chifaa resulted in a higher hardness value compared to blending with Svevo (15.53 N). The couscous produced from the 100% Svevo (control) exhibited the lowest hardness value (11.72 N).

Adhesiveness, defined as the negative force required to detach the compression probe from the sample surface, is generally considered an undesirable attribute from a consumer perspective, as excessive adhesiveness negatively affects product quality. In the present study, couscous samples containing Chifaa exhibited lower adhesiveness values (−0.73 and −0.79), whereas higher adhesiveness values were observed in couscous samples prepared with blends of Svevo (−2.15 and −2.54). The couscous produced from the 100% Svevo (control) exhibited a lower hardness value (−1.70) than its blends. When hardness and adhesiveness are considered together, these results indicate that blending with Chifaa yields couscous with improved textural quality, characterized by higher firmness and lower stickiness. Increased adhesiveness has previously been associated with higher fiber content or greater solubility of product constituents (Boudouira et al. 2023).

Different trends were observed for the remaining textural parameters. Springiness and chewiness were evaluated to assess elasticity, defined as the ability of pasta to recover its original shape after compression, and the chewing effort required, as both parameters are related to resistance during the second compression cycle (Laleg et al. 2016; Texture Technologies Corp 2026). Except for the Chifaa/Lentil Malt sample, couscous samples exhibited comparable springiness values (0.92–0.93). The Chifaa/Lentil Malt sample, however, showed a significantly lower springiness value (0.83) than the other samples (*p* < 0.05). Laleg et al. (2016) similarly reported reduced springiness in legume‐based pasta products, which was attributed to the absence of strong disulfide bonds as the proportion of legume flour increased.

Consistent with the hardness results, the Chifaa/Lentil couscous sample exhibited the highest chewiness value (8.78), likely due to its greater firmness (Boudouira et al. 2023). No significant differences were observed among the remaining samples, whose chewiness values ranged from 6.75 to 7.04 (*p* > 0.05). The couscous produced from the 100% Svevo (control) exhibited the lowest chewiness value (6.66). Previous studies have shown that increases in hardness and chewiness in pasta and couscous products are commonly associated with higher incorporation levels of pulse flours, owing to their elevated protein content (Allan et al. 2025). In the present study, the trend observed for protein content was consistent with that observed for hardness values.

Cohesiveness describes the ability of pasta to stick to itself and maintain structural integrity during compression (Laleg et al. 2016; Tekin‐Cakmak et al. 2024). The cohesiveness values of the couscous samples ranged from 0.52 to 0.58, with no significant differences detected among samples (*p* > 0.05), although the Chifaa/Lentil sample showed a slightly higher value. Resilience, defined as the ability of a product to recover its original height after deformation (Texture Technologies Corp 2026), followed a pattern similar to that observed for springiness. Except for the Chifaa/Lentil Malt and 100% Svevo (control) samples, couscous samples exhibited statistically comparable resilience values (0.35–0.37) (*p* > 0.05), while the lowest resilience was observed in the Chifaa/Lentil Malt sample (0.27). Overall, substituting Lentil Malt for Lentil flour did not markedly affect hardness, adhesiveness, or cohesiveness but resulted in reduced springiness, chewiness, and resilience in the Chifaa/Lentil formulation.

There are no standardized instrumental criteria or reference ranges that have been established for evaluating the textural properties of couscous. This is largely due to the fact that texture profile measurements are strongly affected by factors such as sample preparation, rehydration conditions, and instrumental parameters, which limit direct comparisons across studies. Therefore, the quality of newly developed couscous products is typically evaluated by comparing their textural characteristics with those of semolina couscous and other couscous‐like products reported in the literature. The textural properties obtained in this study are in general agreement with the values reported in the literature. Aboubacar and Hamaker (1999) reported lower hardness (11.7–13.4 N) and chewiness (4.1–4.5) values for commercial and laboratory‐scale durum wheat couscous compared with those observed in the present study (15.53–19.58 N and 6.75–8.78, respectively). In contrast, Yuksel et al. (2017) reported couscous‐like products with substantially higher adhesiveness (−14.10 to −172.16), lower springiness (0.28–0.53), lower cohesiveness (0.30–0.41), lower resilience (0.09–0.16), and markedly higher chewiness values (20.58–91.73). Similarly, Boudouira et al. (2023) evaluated couscous produced from 100% rice flour and reported lower adhesiveness (−6.11), springiness (0.35), cohesiveness (0.37), and resilience (0.17), but higher chewiness (20.55), compared with the samples analyzed in the present study. They also observed wider variability in textural parameters for couscous formulations containing legume flours, showing trends consistent with those observed for rice‐based couscous.

### 3.5 Phenolic Contents and Antioxidant Capacities of Flour and Couscous Samples

Phenolic contents and antioxidant capacities (free, bound, and total) of the flour and couscous samples are presented in Table 5. No significant differences were observed in free, bound, or total phenolic contents between Chifaa and Lentil flours (478.37 and 474.91 mg GAE/100 g, respectively) (*p* > 0.05); however, both flours exhibited significantly higher total phenolic contents than Svevo and Lentil Malt flours (451.17 and 453.25 mg GAE/100 g, respectively) (*p* < 0.05). Among all flour samples, Chifaa flour displayed the highest free (237.65 mg GAE/100 g), bound (240.72 mg GAE/100 g), and total phenolic contents.

**TABLE 5:** Phenolic content and antioxidant capacities (ABTS, FRAP, and DPPH) of the flour and couscous samples.

	Samples	Phenolic contents	ABTS	FRAP	DPPH
Flour					
Free	Svevo	218.91 ± 0.94^c^	99.47 ± 0.65^d^	63.50 ± 0.71^d^	104.11 ± 1.45^d^
	Chifaa	237.65 ± 2.39^a^	269.25 ± 2.71^a^	282.00 ± 3.22^a^	280.89 ± 1.50^a^
	Lentil	236.18 ± 2.24^a^	237.35 ± 4.04^b^	220.42 ± 0.94^b^	265.96 ± 1.53^b^
	Lentil Malt	225.55 ± 1.43^b^	172.10 ± 0.33^c^	112.96 ± 0.55^c^	162.09 ± 1.14^c^
Bound	Svevo	232.26 ± 2.22^B^	164.96 ± 0.65^D^	192.50 ± 1.89^C^	210.11 ± 3.84^C^
	Chifaa	240.72 ± 1.15^A^	275.48 ± 1.35^A^	304.26 ± 1.51^A^	291.48 ± 2.30^A^
	Lentil	238.73 ± 2.25^A^	270.32 ± 2.65^B^	239.00 ± 0.98^B^	278.25 ± 2.94^B^
	Lentil Malt	227.70 ± 0.63^C^	208.14 ± 1.02^C^	193.41 ± 0.96^C^	184.88 ± 1.14^D^
Total**	Svevo	451.17 ± 1.55^y^	264.43 ± 1.13^t^	256.00 ± 1.24^t^	314.22 ± 2.52^t^
	Chifaa	478.37 ± 1.33^x^	544.73 ± 4.06^x^	586.26 ± 2.09^x^	572.37 ± 2.30^x^
	Lentil	474.91 ± 3.59^x^	507.67 ± 6.66^y^	459.42 ± 1.52^y^	544.21 ± 4.43^y^
	Lentil Malt	453.25 ± 2.06^y^	380.24 ± 1.27^z^	306.38 ± 0.55^z^	346.97 ± 1.97^z^
Couscous*					
Free	Svevo (control)	202.84 ± 0.82^d^	108.08 ± 0.87^d^	73.01 ± 0.62	92.10 ± 0.97^e^
	Svevo/Lentil	213.27 ± 1.57^b^	167.90 ± 2.36^c^	168.68 ± 1.11^b^	175.24 ± 2.06^c^
	Svevo/Lentil Malt	207.53 ± 0.54^c^	164.38 ± 0.80^c^	106.46 ± 1.15^c^	110.02 ± 2.49^d^
	Chifaa/Lentil	223.05 ± 2.12^a^	221.37 ± 2.16^a^	234.27 ± 1.35^a^	199.48 ± 1.73^a^
	Chifaa/Lentil Malt	215.64 ± 1.06^b^	196.87 ± 3.34^b^	170.11 ± 2.01^b^	182.73 ± 2.08^b^
Bound	Svevo (control)	210.45 ± 2.40^C^	111.16 ± 0.30^E^	175.12 ± 1.40^D^	152.75 ± 0.48^E^
	Svevo/Lentil	215.62 ± 1.21^B^	206.13 ± 0.94^B^	186.92 ± 1.17^B^	229.26 ± 2.86^A^
	Svevo/Lentil Malt	208.92 ± 1.81^D^	178.03 ± 1.61^D^	178.54 ± 2.06^C^	165.18 ± 2.84^D^
	Chifaa/Lentil	225.51 ± 1.47^A^	230.76 ± 1.42^A^	235.89 ± 2.76^A^	217.09 ± 2.40^B^
	Chifaa/Lentil Malt	218.41 ± 0.59^B^	198.35 ± 1.43^C^	183.94 ± 1.12^B^	191.98 ± 3.71^C^
Total*	Svevo (control)	413.29 ± 3.12^t^	219.24 ± 2.18^t^	248.13 ± 1.82^z^	244.86 ± 1.45^t^
	Svevo/Lentil	428.89 ± 1.74^y^	374.03 ± 2.66^y^	355.60 ± 2.26^x^	404.49 ± 1.05^x^
	Svevo/Lentil Malt	416.46 ± 2.13^z^	342.41 ± 0.84^z^	285.00 ± 2.67^y^	273.78 ± 3.91^z^
	Chifaa/Lentil	448.56 ± 1.84^w^	452.12 ± 3.27^w^	470.16 ± 4.10^w^	416.57 ± 3.70^w^
	Chifaa/Lentil Malt	434.04 ± 1.65^x^	395.22 ± 3.44^x^	354.05 ± 0.93^x^	374.72 ± 3.61^y^

*Note:* Data are expressed as mean ± standard deviation. Different letters within the same column indicate significant differences for the same fraction (free; a–d, bound; A–D, total; w–t) of different samples, according to Tukey's post hoc test (*p* < 0.05). Grain samples (wheat, lentil, and barley) and couscous samples were evaluated separately.

* Svevo or Chifaa was mixed with Lentil or Lentil Malt in equal proportions before grinding. Phenolic contents are expressed as mg GAE/100 g dry weight (dw). ABTS: 2,2′‐azino‐bis (3‐ethyl‐benzothiazoline6‐sulphonic acid); DPPH: 2,2‐diphenyl‐1‐picrylhydrazyl radical scavenging activity; FRAP: Ferric reducing antioxidant power.

** The sum of free and bound antioxidant capacities expressed as mg TE/100 g dw.

Antioxidant capacity was measured using three assays. These assays are based on different reaction mechanisms and exhibit varying sensitivities toward different classes of antioxidant compounds. DPPH and ABTS often exhibit stronger correlations because both evaluate radical scavenging activity, whereas FRAP measures reducing power. The combined application of these assays provides complementary information and allows a more comprehensive assessment of the antioxidant potential of food samples. Consistent with total phenolic content results, antioxidant activity evaluated using three different assays revealed that Chifaa flour exhibited the highest free, bound, and total antioxidant capacities among the flour samples. In contrast, malting significantly reduced the phenolic contents and antioxidant activities of lentil flour (*p* < 0.05). This behavior was previously observed by Ikram et al. (2021), who reported a decline in soluble TPC, including sprouted soybean and lentil, mung bean, and black bean. The free, bound, and total phenolic contents and ABTS values of Chifaa flour were comparable to those previously reported for Chifaa flour used in bulgur production (Tekin‐Cakmak et al. 2024). However, Chifaa flour used for bulgur production showed higher antioxidant activity as determined by DPPH (294.08, 399.6, and 693.7 mg TE/100 g dw).

A similar trend was observed in couscous samples. Formulations containing Chifaa generally exhibited higher phenolic contents than those prepared with Svevo flour. The highest free, bound, and total phenolic contents were recorded for the Chifaa/Lentil couscous sample, with values of 223.05, 225.51, and 448.56 mg GAE/100 g dw, respectively. The lowest free, bound, and total phenolic contents were found for the 100% Svevo (control) couscous sample. Accordingly, this sample also showed the highest total antioxidant capacities across all assays, reaching 416.57 mg TE/100 g (DPPH), 470.16 mg TE/100 g (FRAP), and 452.12 mg TE/100 g (ABTS).

Incorporation of Chifaa barley into the couscous formulation, therefore, markedly enhanced phenolic contents and antioxidant capacities compared to traditional couscous, while also resulting in the lowest glycemic index and the highest β‐glucan content, as discussed in the previous section. These findings support the hypothesis that foods rich in phenolic compounds and dietary fiber may reduce starch hydrolysis and attenuate glycemic response. This effect is likely related to the ability of phenolic compounds to inhibit digestive enzymes by interacting with starch‐degrading enzymes, thereby slowing starch hydrolysis (Khemiri et al. 2022).

In contrast, the Svevo/Lentil couscous sample exhibited free and bound phenolic contents and antioxidant activities comparable to those of the Chifaa/Lentil Malt sample, and even higher antioxidant activity when assessed by DPPH (*p* > 0.05). Similarly, the 100% Svevo (control) sample exhibited the lowest total antioxidant capacities, measured as 219.24 mg TE/100 g (ABTS), 248.13 mg TE/100 g (FRAP), and 244.86 mg TE/100 g (DPPH).

Overall, the phenolic contents and antioxidant activities of couscous samples in the present study were substantially higher than those reported for traditional couscous in several previous studies (Cankurtaran Kömürcü 2025; Yuksel et al. 2017). However, Cankurtaran and Bilgiçli (2021) reported an even higher total phenolic content (587.3 mg GAE/100 g) for traditional couscous, exceeding the range observed in this study (416.5–448.6 mg GAE/100 g dw). Legumes, which are naturally rich in polyphenolic compounds, typically exhibit high antioxidant activity and are therefore regarded as functional foods. Consequently, legume flours have been widely proposed as ingredients for the development of value‐added cereal‐based products (Turco et al. 2019).

Several studies have demonstrated that enrichment of couscous with barley or legume flours, as well as spices, can significantly increase phenolic contents and antioxidant capacities. For example, couscous enriched with pea or mung bean flour combined with ginger or turmeric powders exhibited total phenolic contents ranging from 382.2 to 609.7 mg GAE/100 g and from 365.2 to 602.8 mg GAE/100 g, respectively (Cankurtaran Kömürcü 2025). Oriente et al. (2020) reported that incorporation of 20% and 30% barley coarse fraction into couscous formulation increased total phenolic content by 11‐ and 15‐fold, respectively, compared to control couscous. Similarly, Turco et al. (2019) reported total phenolic contents ranging from 87 to 176 mg GAE/100 g in pasta produced entirely from legume flours, which were higher than those of durum wheat semolina pasta (63.8 ± 7 mg GAE/100 g). Carcea et al. (2017) reported total phenolic contents of 470.0 and 624.8 mg GAE/100 g dw for couscous produced from durum wheat semolina and barley, respectively, values exceeding those observed in the present study. These variations among studies may be attributed to differences in raw material variety, formulation, and analytical methods used for phenolic determination (Oriente et al. 2020).

## 4 Conclusion

This study demonstrates that enriching traditional couscous with hull‐less barley (cv. Chifaa) and lentil‐based ingredients enhances its nutritional and functional quality while preserving its role as a staple food within the Mediterranean diet. The incorporation of locally relevant raw materials, particularly β‐glucan–rich barley and lentils, improved cooking performance (water absorption and volume increase), textural properties (hardness, adhesiveness, and chewiness), antioxidant capacity, and glycemic response of couscous. Couscous formulations containing Chifaa flour exhibited higher β‐glucan, dietary fiber, and protein contents and achieved low in vitro GI values, supporting dietary patterns that emphasize whole grains, legumes, and low‐GI foods, key components of the Mediterranean diet. The increased phenolic content and antioxidant activity further contribute to the health‐promoting characteristics associated with Mediterranean dietary traditions. Overall, the development of couscous enriched with Chifaa barley and lentil‐based ingredients offers a promising approach to improving the nutritional profile of traditional cereal products commonly consumed in Mediterranean food systems. In future studies, the potential for developing a low‐GI functional couscous should be verified through in vivo tests, while the sensory quality and consumer acceptance of couscous samples should also be investigated.

## Author Contributions

**Ece Surek:** writing – review and editing, writing – original draft, data curation. **Kubra Ozkan:** writing – review and editing, investigation, data curation. **Cagla Ozer:** writing – review and editing. **Abderrazek Jilal:** conceptualization, investigation, writing – review and editing. **Ghizlane Salih:** resources, investigation, writing – review and editing. **Halide Yildirim:** investigation, data curation. **Andrea Visioni:** resources, writing – review and editing. **Francesco Sestili:** project administration, conceptualization, writing – review and editing. **Eda Aktas‐Akyildiz:** investigation, writing – review and editing. **Osman Sagdic:** writing – review and editing. **Hamit Koksel:** conceptualization, supervision, writing – review and editing.

## Funding

The research was funded by MEDWHEALTH PRIMA Foundation (Horizon2020), PRIMA SECTION 1 2020 AGROFOOD VALUE CHAIN IA TOPIC: 1.3.1‐2020 (IA), MEDWHEALTH project grant no. 2034.

## Ethics Statement

This study does not involve any human or animal testing.

## Conflicts of Interest

The authors declare no conflicts of interest.

## Data Availability

The data that support the findings of this study are available on request from the corresponding author. The data are not publicly available due to privacy or ethics restrictions.
